# Effects of histone acetylation on superoxide dismutase 1 gene expression in the pathogenesis of senile cataract

**DOI:** 10.1038/srep34704

**Published:** 2016-10-05

**Authors:** Xianfang Rong, Xiaodi Qiu, Yongxiang Jiang, Dan Li, Jie Xu, Yinglei Zhang, Yi Lu

**Affiliations:** 1 Department of Ophthalmology, Eye and ENT Hospital of Fudan University, Shanghai, China.; 2 Key Laboratory of Myopia of State Health Ministry, and Key Laboratory of Visual Impairment and Restoration of Shanghai, Shanghai, China

## Abstract

Histone acetylation plays key roles in gene expression, but its effects on superoxide dismutase 1 (SOD1) expression in senile cataract remains unknown. To address this problem, the study was to investigate the influence of histone acetylation on SOD1 expression and its effects in the pathogenesis of senile cataract. Senile cataract was classified into three types—nuclear cataract (NC), cortical cataract (CC), and posterior subcapsular cataract (SC)—using the Lens Opacities Classification System III. In senile cataracts, SOD1 expression decreased significantly. Both H3 and H4 were deacetylated at −600 bp of the SOD1 promoter of cataract lenses, and hypoacetylated at −1500, −1200, and −900 bp. In hypoacetylated histones, the hypoacetylation pattern differed among the cataracts. *In vitro*, anacardic acid (AA) significantly reduced H3 and H4 acetylation at the SOD1 promoter, decreased protein expression, and induced cataract formation in rabbits. AA also inhibited HLEC viability and increased cell apoptosis. In contrast, trichostatin A (TSA) was able to efficaciously stop AA’s effects on both rabbit lenses and HLECs. Decreased histone acetylation at the SOD1 promoter is associated with declined SOD1 expression in senile cataracts. Histone acetylation plays an essential role in the regulation of SOD1 expression and in the pathogenesis of senile cataracts.

Cataract remains the leading cause of visual disability and blindness worldwide^1^. While surgical intervention is currently the most effective remedy^2^, the high incidence rate of the disease and the high cost of surgery present a great economic burden among developing countries^1^. In addition, there is a greater chance of irreversible sight-threatening surgery-associated complications^3^,^4^,^5^. Thus, great emphasis is being placed on finding alternative pharmacological strategies to prevent or delay cataract formation among the elderly population.

Oxidative stress has long been recognized as an initiating factor in the pathogenesis of cataracts^6^,^7^. An imbalance among oxidative stress, antioxidant protection, and repair processes results in the development of cataracts^8^,^9^. The main enzymatic antioxidants in the lens include superoxide dismutase (SOD), catalase, and glutathione peroxidase^10^. SOD provides the first line of defense against deleterious reactive oxygen species (ROS). Three isoforms of SOD—cytosolic SOD1, SOD2, and SOD3—have been identified; SOD1 is the major isoform in the lens and accounts for nearly 90% of total SOD activity in the lens^11^. Studies have revealed that animals lacking this specific enzyme show signs of premature aging, such as accelerated hearing loss^12^, neurological degeneration^13^, age-related macular degeneration^14^, shortened lifespan^15^, and development of enhanced age-related cataract at an earlier age^16^. It has been reported that specific SOD1 activity declines as a function of age in human lenses^6^,^7^ and in the blood of cataract patients^17^. Studies have also suggested that the decreased activity of the SOD1 isoform in cataractous lenses is associated with the decreased level of mRNA transcripts and their protein expression^18^. Overexpression of SOD1 has been shown to prevent ROS-induced oxidative damage to lenses and to control cataract formation^19^. Impaired SOD1 activity in cataractous lenses might result from genetic polymorphisms of the SOD1 genes^20^, mutations in coding and noncoding regions^18^, and epigenetic regulation through promoter modifications^21^. However, the precise mechanisms of the decreased expression of SOD1 in senile cataractous lenses have not been well established yet.

Histone covalent modifications, including acetylation, methylation, and phosphorylation, alter the configuration of chromatin for interaction with transcription factors and the basal transcriptional machinery^22^. Among the modifications, histone acetylation has been studied extensively, and it occurs mainly in the promoter region of transcribed genes. Enhanced transcription is often correlated with increased histone acetylation^23^,^24^, whereas silencing is commonly associated with histone hypoacetylation^25^,^26^. Histone acetylation is a dynamic process controlled by histone acetyltransferases (HATs), which catalyze histone acetylation, and histone deacetylases (HDACs), which catalyze histone deacetylation^22^. HDAC inhibitors, which block the effects of HDAC, can also enhance histone acetylation, thereby altering transcription^27^. Among the four core histone proteins that comprise the central chromatin core (H2A, H2B, H3, and H4), acetylation processes on H3 and H4 seem particularly important in gene regulation^28^. Due to the intimate relationship between histone acetylation and gene expression, multiple HAT and HDAC inhibitors have been studied and even undergone clinical trials to attempt to disrupt the balance between HATs and HDACs^27^,^29^,^30^.

To date, no studies have been conducted to determine whether histone acetylation regulates SOD1 expression in senile cataracts and whether the regulation is associated with the development of cataracts. Thus, the present study was designed to explore histone acetylation and SOD1 expression changes in the lens epithelia of senile cataract cases and to illustrate the regulation effects of histone acetylation modification on SOD1 gene expression in the pathogenesis of senile cataracts.

## Results

### General Characteristics of Patient Samples

The type of cataract was categorized, using the Lens Opacities Classification System III, into the following subtypes: nuclear cataract (NC), cortical cataract (CC), and posterior subcapsular cataract (SC). The general characteristics of the patients in each group are summarized in Table 1. There were 40 total samples in the NC, CC, and SC groups and 12 in the control group respectively. The mean ages of the patients were 74.9 ± 7.6 years in the NC group, 70.2 ± 9.9 years in the CC group, 71.4 ± 9.1 years in the SC group, and 35.3 ± 2.8 years in the control group. The axial lengths were 22.96 ± 0.76 mm in the NC group, 23.07 ± 0.77 mm in the CC group, 23.08 ± 0.60 mm in the SC group, and 22.89 ± 0.64 mm in the control group.

### Acetylation Level of H3 and H4

The genome-wide histone acetylation levels of H3 and H4 in the anterior lens epitheliums (LEs) of senile cataract patients were analyzed with the Western blot method. As showed in Supplementary Figure S1, although a slight decrease in H3 and H4 acetylation was detected across the genome in the NC, CC, and SC patients, there were no significant differences when compared with the normal controls.

**Figure 1:**
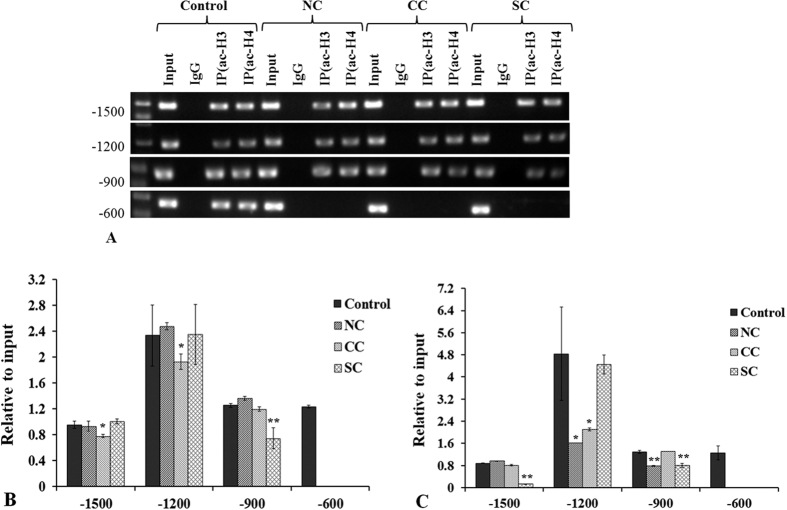
CHIP-PCR analysis of H3 and H4 acetylation in SOD1 promoter of senile cataracts. (**A**) Both H3 and H4 were deacetylated at −600 bp of three types of senile cataracts. (**B**) Decreased acetylation of H3 occurred at −1500 and −1200 bp of the SOD1 promoter in the CC group and at −900 bp in the SC group. (**C**) Decreased H4 acetylation was detected at −1200 and −900 bp in the NC group, at −1200 bp in the CC group, and at −1500 and −900 bp in the SC group. *p < 0.05; **p < 0.001.

Differences in acetylated H3 and H4 positions in the promoter region of SOD1 were detected by chromatin immunoprecipitation (CHIP)- real-time polymerase chain reaction (PCR) assay, and the acetylation level was confirmed by quantitative PCR (qPCR). The SOD1 promoter region −3000~0 bp was analyzed. The CHIP-PCR results showed that acetylation of both H3 and H4 was detected at −1500, −1200, −900, and −600 bp of the SOD1 promoter in the control LEs. In the LEs of the NC, CC, and SC patients, the acetylated positions were at −1500, −1200, and −900 bp, respectively (Fig. 1A).

QPCR of the precipitated DNA regions by CHIP revealed that although no differences in acetylated positions were found among the three types of cataract, the acetylation level varied at different acetylated regions (Fig. 1B,C). There were no significant differences in acetylated H3 at −1500 bp in the NC and SC groups compared with the controls (p = 0.63 and 0.24, respectively); however, the acetylation level in the CC group decreased remarkably (p = 0.003). Similar results were detected at −1200 bp. At −900 bp, there were no significant differences in acetylated H3 in the NC and CC groups compared with the controls (p = 0.15 and p = 0.43, respectively); however, the acetylation level in the SC group decreased remarkably (p < 0.001) (Fig. 1B). The acetylated H4 at −1500 bp decreased significantly in the SC group (p < 0.001), but there were no significant changes in the NC (p = 0.06) and CC (p = 0.05) groups. Acetylated H4 at −1200 bp was significant lower in the NC and CC groups than that in the control group (p = 0.002 and p = 0.005, respectively); however, no difference was found between the SC and control groups (p = 0.60). Different H4 acetylation results were found at −900 bp, with significant reductions in acetylation level in the NC (p < 0.001) and SC (p < 0.001) groups and no change in the CC group (p = 0.50) (Fig. 1C).

**Figure 2:**
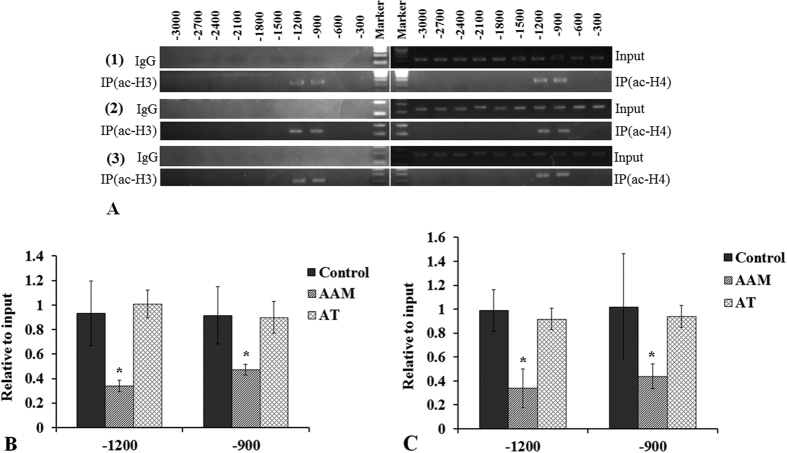
CHIP-PCR analyses of H3 and H4 acetylation in the SOD1 promoter of cultured rabbit lenses treated with AA (AAM group) and AA+TSA (AT group). (**A**) AA did not change the histone acetylation position at the SOD1 promoter. The acetylated histones were at −1200 and −900 bp in all of the groups (A(1) control group; A(2) AAM group; A(3) AT group). (AA significantly reduced histone acetylation at both −1200 and −900 bp ((**B**) H3 relative acetylation level; (**C**) H4 relative acetylation level). TSA effectively prevented histone hypoacetylation induced by AA. *p < 0.05.

The influences of HAT inhibitor anacardic acid (AA) and HDAC inhibitor trichostatin A (TSA) on rabbit LE H3 and H4 acetylation were also analyzed by CHIP-PCR. The results revealed that at −3000~0 bp of the SOD1 promoter region, acetylation was only detected at −1200 and −900 bp in both H3 and H4 in the control. The acetylation positions of H3 and H4 kept the same with the control at the SOD1 promoter in both AAM and AT group (Fig. 2A). The results of the qPCR analysis revealed that AA reduced the acetylation levels of H3 and H4 significantly at −1200 and −900 bp (all p < 0.05). TSA prevented AA from having an effect on H3 and H4 acetylation, as shown in Figs 2B and 2C of AT group; p > 0.05 compared with the control.

### Expression Level of SOD1

**Figure 3:**
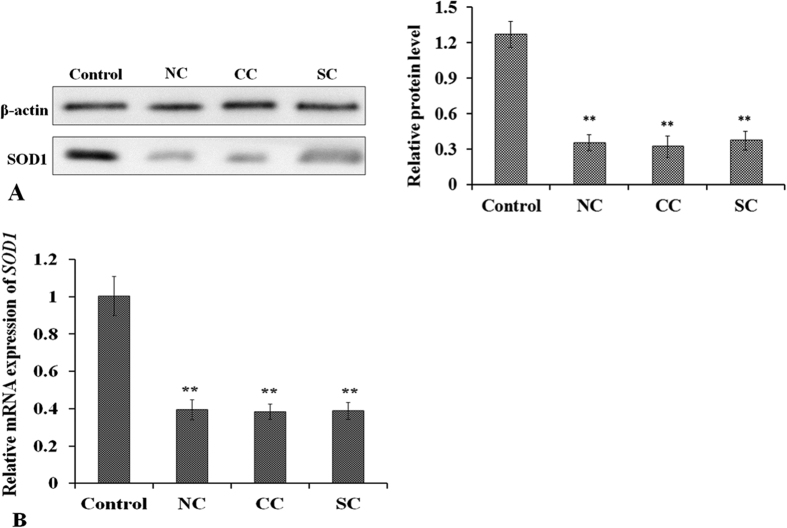
Western blot (**A**) and qPCR (**B**) analysis of SOD1 expression in senile cataracts. (**A**) SOD1 expression was lower in all cataract types—NC, CC, and SC—compared with the controls. (**B**) mRNA level of SOD1 was also significantly lower in all types of cataracts. **p < 0.001.

To evaluate SOD1 expression changes in senile cataract LEs, Western blot and qPCR were used. As shown in Fig. 3, the expression level of SOD1 decreased significantly in NC, CC, and SC LEs, with all p < 0.001 in both protein and mRNA level compared with the control group. No differences in SOD1 expression were detected among the three types of cataract at the protein and mRNA level.

**Figure 4:**
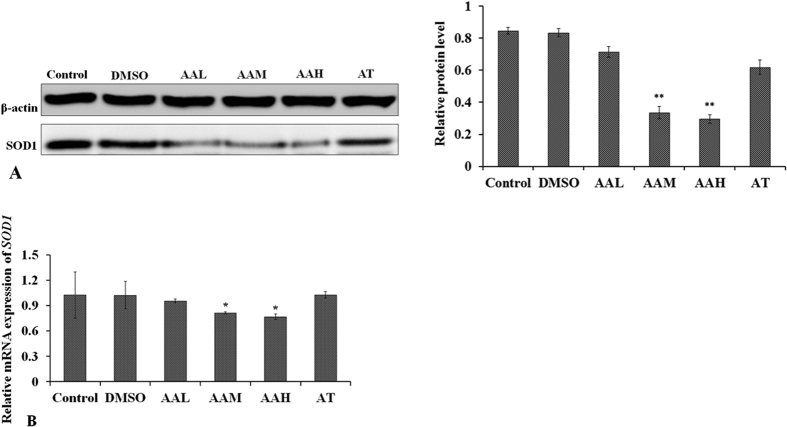
Western blot (**A**) and qPCR (**B**) analysis of changes in SOD1 expression in the rabbit lenses *in vitro* treated with different concentrations of AA and AA+TSA. After incubation for three weeks, (**A**) *SOD1 protein* expression decreased in the AA-treated lenses and (**B**) the mRNA levels of *SOD1 decreased significantly in the AAM and AAH groups.* No differences in SOD1 expression were found in the AT group compared with the controls. *p < 0.05, **p < 0.001.

**Figure 5:**
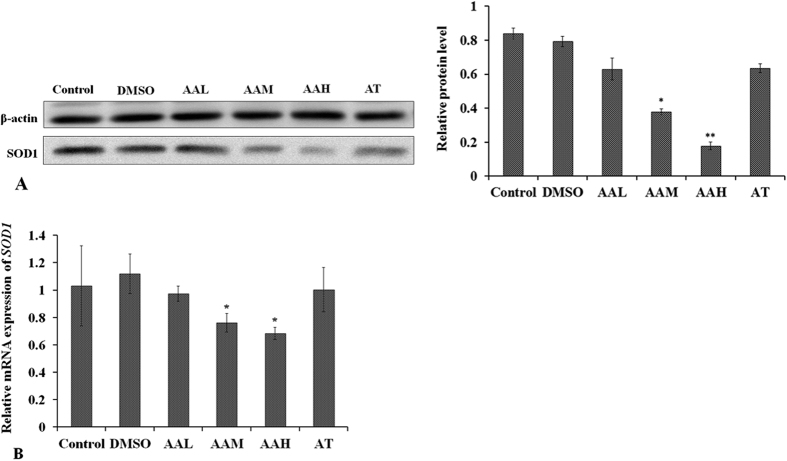
Western blot (**A**) and qPCR (**B**) analysis of changes in SOD1 expression in HLECs treated with different concentrations of AA and AA+TSA. After being treated for 48 hours, (**A**) *SOD1 protein* expression decreased in HLECs supplemented with AA in the AAM and AAH groups and (**B**) the mRNA levels of *SOD1 decreased significantly in the AAM and AAH groups.* No differences in SOD1 expression were found in the AA+TSA-treated HLECs compared with the controls. *p < 0.05, **p < 0.001.

SOD1 expression influenced by AA or TSA was explored by rabbit lens and HLEC culture. The results suggested that SOD1 protein expression was reduced significantly by moderate (AAM, p < 0.001), and high concentrations (AAH, p < 0.001) of AA in both rabbit lens LEs and HLECs (Figs 4A and 5A). mRNA levels were also reduced in the AAM (p = 0.04) and AAH (p = 0.03) groups (Figs 4B and 5B). TSA was able to prevent AA’s influence on SOD1 expression at the protein and mRNA levels in AT group, as shown by Western blot and qPCR (Figs 4 and 5).

Immunofluorescence staining of the cultured HLECs revealed that SOD1 was expressed mainly in the cytosol and nuclei of the HLECs. Remarkably weakened staining intensity of SOD1 was seen in the AAM and AAH groups compared with the control (Supplementary Figure S4). In the AT group, an almost control-like staining of SOD1 was detected. It is suggested that the effects of TSA might counteract the influence of AA on SOD1 expression.

### Rabbit Lens Culture and Cataract Formation

To examine whether long-term culture of transparent lenses in the presence of AA would lead to lens opacity formation and the development of cataracts, and whether TSA could prevent this process, rabbit lenses were cultured for up to three weeks. Lens clarity was monitored every day. Lenses cultured for one week in the presence of AA developed opacity in a dose-dependent manner; transparency decreased as concentration of AA increased. Lens opacity remained stable at the end of the second week. However, lenses cultured with AA+TSA maintained almost the same level of clarity as lenses in the DMSO and control groups, which remained nearly transparent except for opacification that developed in the equatorial area (Supplementary Figure S5).

### HLEC Viability

**Figure 6:**
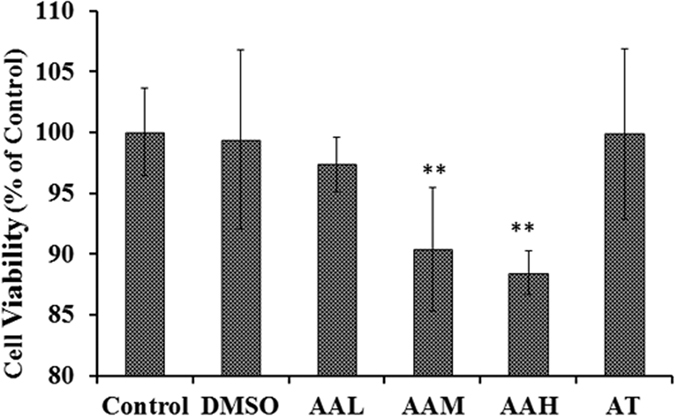
CCK-8 assay showed that HLEC viability decreased significantly in the AAM and AAH groups. TSA prevented the effect of AA on HLECs, as shown in the AT group figure. *p < 0.05.

The effects of different concentrations of AA on the viability of HLECs were evaluated by Cell Counting Kit-8 (CCK-8) assay. The results showed that HLEC viability decreased significantly in the AAM and AAH treated groups (both p < 0.001). However, cell viability in the AT group remained similar (p = 0.96) to that of the control group, as shown in Fig. 6. These findings suggest that TSA prevents AA from affecting the viability of HLECs.

### HLEC Apoptosis

**Figure 7:**
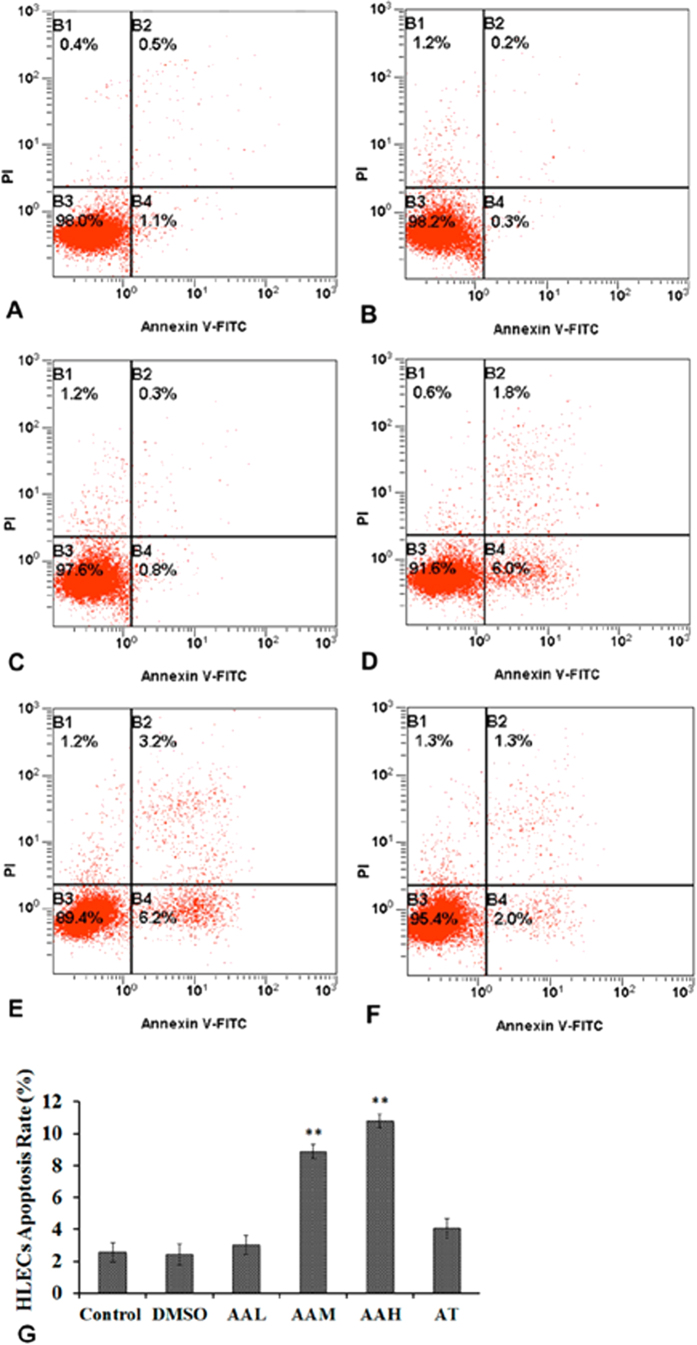
Flow cytometry analyses of HLEC apoptosis induced by AA after being cultured for 48 hours. The cell apoptosis rate increased remarkably in the AAM and AAH groups. The apoptosis rate of the A+T group did not different significantly from that of the control group. (**A**) control group; (**B**) DMSO group; (**C**) AAL group; (**D**) AAM group; (**E**) AAH group; (**F**) AT group.

To determine whether AA could induce HLEC apoptosis and whether TSA could prevent the effects of AA, flow cytometry was used to detect the apoptosis rate at 48 hours after cell culture. The apoptosis rates of the control and DMSO groups were 2.57 ± 0.60% and 2.40 ± 0.66%, respectively (Fig. 7). The AAL group had an apoptosis rate of 3.00 ± 0.60%, with p = 0.37 compared with the controls. However, in the AAM and AAH groups, the apoptosis rate increased to 8.87 ± 0.45% and 10.80 ± 0.44%, respectively, both p < 0.001 compared with the controls. TSA significantly protected the HLECs from apoptosis induced by AA, as shown in the AT group, with an apoptosis rate of 3.01 ± 0.60%.

## Discussion

Previous studies have reported that histone acetylation is generally found to be associated with gene activation^31^, while silenced and repressed genes are thought to be stably hypoacetylated^32^. Acetylation occurs mainly in the promoter region of transcribed genes. HDAC inhibitors, HATs, HDACs, and HAT inhibitors, which are related to histone hyper- and hypoacetylation, have the effects of gene activation or repression^31^. Enhanced acetylation of H3 and H4, which are core histones of nucleosomes, plays an essential role in remodeling chromatin and converting genes to an active state^23^. However, to the best of our knowledge, the regulation effects of histone acetylation in the pathogenesis of senile cataract have not yet been reported.

The aim of the present study was to explore differences in histone acetylation modification at the SOD1 promoter region between normal transparent lenses and senile cataracts. The results showed that both H3 and H4 were deacetylated at −600 bp of SOD1 promoter in the LE of senile cataracts. Changes in the hypoacetylated region also varied among the different types of cataracts. Consistent with the decreased level of histone acetylation was reduced SOD1 expression at both the protein and mRNA levels. The *in vitro* study suggested that reducing histone acetylation with a HAT inhibitor could decrease SOD1 expression and induce cataract formation. The HDAC inhibitor effectively prevented the cataract formation effect of the HAT inhibitor. The results provided evidence that histone acetylation plays an important role in regulating SOD1 expression and in the pathogenesis of senile cataracts.

SOD1, which accounts for nearly 90% of total lens SOD activity^11^, provides the first firewall in defense against ROS. In cells, ROS are the primary source of oxidative damage, which has long been recognized as an initiating factor in the pathogenesis of cataracts. Reduced SOD1 expression in lenses has led to premature cataract formation in animal studies^33^, and overexpression of SOD1 in lenses has prevented the development of cataracts induced by oxidative damage^19^. In senile cataract patients, both the expression level and enzyme activity of SOD1 have been shown to decrease significantly^18^.

In the present study, the results showed that SOD1 protein level was much lower in all three types of cataracts than in clear normal lenses, which is consistent with the findings of previous studies^18^. The mRNA expression of SOD1 was also reduced, as showed in Fig. 3. Correspondingly, the results revealed that both H3 and H4 were deacetylated at −600 bp of the SOD1 promoter of cataract lenses, and decreased histone acetylation at the SOD1 promoter was detected at −1500, −1200, and −900 bp. However, in hypoacetylated histones, the decreased histone acetylation pattern differed among the three types of cataracts (Fig. 1B,C). In the senile cataract patients, decreased acetylation of H3 occurred at −1500 and −1200 bp in the CC group and at −900 bp in the SC group. Decreased acetylation of H4 occurred at −1200 and −900 bp in the NC group, at −1200 bp in the CC group, and at −1500 and −900 bp in the SC group. Based on these results, it can be presumed that histone acetylation at −600 bp of SOD1 promoter might be the key to the regulation of SOD1 expression and histone deacetylation at −600 bp might contribute to the downregulation of SOD1 expression and the formation of cataracts. While the differences in histone acetylation among the NC, CC, and SC groups might be one of the factors responsible for the direction of different types of senile cataracts, the presumptions need verification; the exact mechanisms and functions regarding differences in histone acetylation in the regulation of NC, CC, and SC formation require further investigation.

To confirm that histone acetylation regulates SOD1 expression and cataract formation, the effects of a HAT inhibitor, AA, and an HDAC inhibitor, TSA, were evaluated in the lens and HLEC cultures. AA is a cell-permeable, potent, broad-spectrum, non-competitive inhibitor of HATs with three main classes: GCN5/PCAF, p300/CBP, and the MYST family^34^. TSA is a classical, nonselective, noncompetitive HDAC inhibitor. The results of the current study showed that AA significantly reduced histone acetylation at the SOD1 promoter in rabbit lenses. Simultaneously, SOD1 expression was also downregulated. More importantly, the rabbit lens cultures treated with AA developed cataracts in a concentration-dependent manner. However, lenses treated with AA+TSA in the AT group remained nearly transparent, and SOD1 expression level remained similar to that of the control. The development of cataracts after AA intervention might be due to decreased SOD1 expression and, consequently, increased oxidative insults caused by altered histone acetylation. In the AA+TSA treated lenses, TSA corrected the imbalance effect of HATs and HDACs interrupted by AA and reversed histone acetylation, which resulted in the normal expression of SOD1. TSA also inhibited cell proliferation and migration, and it reversed epithelial–mesenchymal transition (EMT)^35^. In lens epithelial explants, TSA prevented the formation of posterior capsule opacification^36^. Therefore, in the current study, another reason for the transparency of lenses treated with AA+TSA might be TSA’s antiproliferative, antimigrative, and anti-EMT effects on the HLECs.

In lenses, SOD1 is mainly present in the epithelium and in the outer cortex, where cell organelles are present^37^. It is distributed in cells of the cytosol, the nucleus, and the mitochondrial intermembrane space^38^ (Supplementary Figure S4). The special distribution of SOD1 in cells might help it to efficaciously eliminate ROS produced during cellular metabolism and to maintain the physiological functions of the cells. In HLEC cultures treated with AA, SOD1 expression was downregulated through histone deacetylation and hypoacetylation at the SOD1 promoter. The changes altered the metabolic processes of the HLECs and increased oxidative damage, which in turn resulted in decreased HLEC viability and increased cell apoptosis rates. TSA significantly prevented the effects of AA on histone hypoacetylation and the downregulation of SOD1 (Fig. 5). The results further proved that by regulating SOD1 expression, histone acetylation influenced cataract formation and HLEC function.

Limitations were inevitably present in the current study. For example, although histone acetylation changes were detected at −1500~0 bp of the SOD1 promoter region in senile cataracts, the exact acetylated sites in the histones were not determined. In addition, the functions of the differences in histone acetylation in the formation of different types of senile cataracts remained unclear. It is possible that histone acetylation regulates not only SOD1 expression in senile cataracts, but also SOD1 activity changes due to age and cataract formation, as mentioned previously. All of these issues will be the subjects of future investigations.

In conclusion, H3 and H4 acetylation decreased significantly at the SOD1 promoter in senile cataracts. Coincident with the decreased histone acetylation was the downregulated expression of SOD1. The regulation of histone acetylation on SOD1 expression plays an essential role in the pathogenesis of senile cataracts.

## Materials and Methods

The present study adhered to the tenets of the Declaration of Helsinki and received approval from the Ethics Committee of the Eye and ENT Hospital of Fudan University, Shanghai, People’s Republic of China, to collect and use the anterior LEs from patients undergoing cataract surgery. Written informed consent was obtained from the patients before the samples were collected. All animal procedures complied with the Association for Research in Vision and Ophthalmology Statement for the Use of Animals in Ophthalmic and Vision Research and all experimental protocols were reviewed and approved by the Ethics Committee for Animal Care and Use of Fudan University, China.

### Patients and Samples

Each cataract patient aged ≥ 50 years was examined clinically and diagnosed by three experienced ophthalmologists, independently and simultaneously. Patients with primary cataracts and normal axes (21–23.99 mm) admitted to our clinic between September 2013 and September 2014 were included in the study. Patients with uveitis, glaucoma, previous ocular trauma and/or surgeries, other ocular diseases, systemic diabetes, or malnutrition were excluded.

Anterior LE samples (approximately 5 mm) were obtained by continuous curvilinear capsulorhexis during cataract surgery by the same experienced surgeon (YL). Normal clear lens LE samples were collected as controls from donated eyes with normal axial lengths from the Eye Bank of Eye and ENT Hospital; the LE samples were extracted within six hours of donor death.

### Lens and Lens Epithelium Cell Culture

Normal New Zealand rabbits were euthanized by systemic anesthetization with ketamine (40 mg/kg body weight) followed by an intravenous injection of air. Intact lenses were harvested under sterile conditions and pre-incubated in Dulbecco’s modified Eagle’s medium (DMEM; Life Technologies, Grand Island, NY) containing 200 units/ml penicillin (Life Technologies) and 200 μg/ml streptomycin (Life Technologies) for 30 minutes, then cultured in DMEM containing 10% fetal bovine serum (FBS), 100 units/ml penicillin, and 100 μg/ml streptomycin at 37 °C in a 5% CO_2_ atmosphere. Lenses that developed opacification in the first 24 hours were discarded.

B-3 HLECs, as described previously^39^, were obtained from the American Type Culture Collection (Rockville, MD) and cultured in DMEM containing 10% FBS, 100 units/ml penicillin, and 100 μg/ml streptomycin at 37 °C in a humidified 5% CO_2_ atmosphere.

AA (Sigma, St. Louis, MO), a HAT inhibitor, and TSA (Sigma), an HDAC inhibitor, were used in the lens and HLEC cultures. After 24 hours of incubation, the cultured lenses and HLECs were divided into six groups according to final concentrations of AA or AA and TSA supplemented in the culture medium: (1) control group, no AA or TSA added; (2) DMSO group, 1 μl/ml of DMSO, used to dissolve AA or TSA; (3) AAL group, 2 μg/ml of AA in the medium; (4) AAM group, 4 μg/ml of AA in the medium; (5) AAH group, 8 μg/ml of AA in the medium; (6) AT group, 4 μg/ml of AA and 2 ng/ml of TSA in the medium. The DMEM used for lenses and HLEC culture was refreshed every 48 hours. The rabbit lenses were observed every day, and images were taken under a stereo microscope (Leica M165 FC, Germany) after the lenses were cultured for three weeks. Then, the rabbit anterior LE samples were collected. The HLECs were harvested after 48 hours of culture treated with AA or TSA.

### Western Blot Assay

The LE samples were pooled and homogenized at 4 °C for 30 minutes by adding 100 μl of cold radioimmunoprecipitation assay (RIPA). The samples included 24 pieces for each type of cataract LE, six samples of LE in the control group and six samples for each group of rabbit LE. The supernatants were obtained by centrifugation at 12000 g for 10 minutes. The HLECs were harvested and lysed by RIPA, and the supernatants were collected. Proteins of all supernatants were separated by 5% and 12% sodium dodecyl sulfate-polyacrylamide gel electrophoresis under the same conditions and then transferred to polyvinylidene difluoride membranes (Millipore, Bedford, MA) at 300 mA for one hour. The membranes were blocked in 5% bovine serum albumin (BSA) (Sigma) at room temperature (RT) for one hour, followed by incubation with primary antibodies of acetylated histone H3 (ac-H3, 1:500; Millipore, Burlington, MA), ac-H4 (1:500; Millipore), SOD1 (1:500; Abcam, Boston, MA) and β-actin (1:500; Abcam) at 4 °C overnight. Then, the membranes were washed and incubated with horseradish peroxidase-conjugated secondary antibodies (1:5000; Abmart, Shanghai, China) for one hour at RT. Images were developed with an Enhanced Chemiluminescence Detection System (Thermo Scientific, Rockford, USA) under the same condition and the protein bands were quantitatively analyzed with Image J analysis software. The experiment was repeated for at least three times.

### Quantitative Real-Time PCR

QPCR was performed to evaluate the mRNA expression of SOD1 in different samples. Six samples of each type of cataract LE, three control samples and three rabbit samples for each group, were pooled and washed with sterile, homogenized phosphate buffered saline (PBS), pH 7.4. Total RNA of the cataract LEs, rabbit LEs, and HLECs were extracted using an RNeasy Mini Kit (Qiagen, Valencia, CA) according to the manufacturer’s instructions. The procedure of cDNA synthesis from RNA samples was performed with a thermal cycler (Thermal Technology, Santa Rosa, CA) using Oligo dT Primer and Random 6-mers according to the manufacturer’s instruction (PrimeScript RT Reagent Kit; Takara, Osaka, Japan). Real-time PCR reactions were performed with SYBR Premix Ex Taq (Takara), and *β-actin* was used as an endogenous control gene. The primer sequences for gene amplification are summarized in Supplementary Table S1. Changes in the expression of SOD1 were determined by the comparative CT method (2^−ΔΔCT^) of relative quantification, using Vii 7 Software (Life Technologies, Pleasanton, CA). All experiments were performed in triplicate.

### Chromatin Immunoprecipitation (CHIP)-PCR Assay

CHIP experiments were performed using a CHIP assay kit (Millipore, Burlington, MA) according to the manufacturer’s protocols. Briefly, ten samples for each type of cataract LE, three samples of LE for the cataract control, and three samples of rabbit LE for each group were pooled, and proteins and DNA were cross-linked after the samples were fixed with 1% formaldehyde in PBS. The reactions were terminated with glycine and the samples were washed twice with cold PBS. Then, the samples were lysed in 1% sodium dodecyl sulfate, and the DNA was cut into small fragments (300 bp) by sonication. The protein–DNA complexes were precipitated using normal immunoglobulin G, ac-H3 (Millipore) and ac-H4 (Millipore) antibodies. Ten percent of the supernatant fraction from the sheared cross-linked DNA lacking primary antibody was saved as the input. The precipitated DNA was subjected to real-time PCR using primers (Supplementary Table S2) designed for indicated sites in the promoter regions of SOD1 to determine the positions of acetylated histone H3 and H4. Then, the precipitated regions in the SOD1 promoter were quantitatively analyzed by qPCR. The CT value of the CHIP signals detected by qPCR was converted to the percentage of each CHIP signal for input DNA, which was calculated by the ΔΔ method, where the difference of one CT value between the CT value of a CHIP signal and the input signals was calculated as a two-fold change.

### Immunofluorescence

Six wells for each group of HLECs were prepared for immunofluorescence assay. The HLECs were cultured on glass coverslips and washed with PBS, fixed for 10 minutes using freshly prepared 4% formaldehyde in PBS, and permeabilized for 10 minutes with 0.5% Triton X-100 in PBS. The cells were then blocked with 1% BSA at RT for one hour and sequentially incubated with primary anti-SOD1 antibody (1:200; Abcam) at 4 °C overnight and fluorescent dye-conjugated secondary antibody (1:500; Life Technologies) at RT for one hour, with three PBS washes after each step. The HLECs were stained with 1 μg/mL DAPI in methanol for 5 minutes to label the cell nuclei, mounted on glass slides, and imaged using a confocal microscope.

### CCK-8

HLEC cell viability was assessed with a CCK-8 as described by the manufacturer (Dojindo Laboratory, Kumamoto, Japan). Equal numbers of HLECs were seeded onto 96-well plates and incubated in DMEM with a final volume of 100 μl per well. After the samples were treated with AA or TSA as indicated previously, 10 μl of kit reagent were added and the samples were incubated for an additional 2 hours at 37 °C. Absorbance at 450 nm was determined on a multi-well plate reader (Benchmark plus™; Bio-Rad, Tokyo, Japan). Percentage of viable cells was calculated using the following formula:


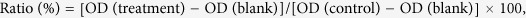


where blank was the culture medium without cells. Each sample was assayed with at least six replications per assay; all experiments were performed at least three times.

### Flow Cytometry

The influence of AA or TSA on HLEC apoptosis was detected with an Annexin-V Fluorescein Isothiocyanate (FITC) Apoptosis Detection Kit I (BD Biosciences, San Diego, CA) according to the manufacturer’s instruction. After treatment, the HLECs were deplated and dispersed with 0.25% trypsin-0.02% EDTA into a single cell suspension and then washed twice with cold PBS and suspended in binding buffer containing 5 μl Annexin V- FITC and 5 μl propidium iodide. After incubating the samples for 15 minutes at RT in the dark, cell apoptosis was analyzed using Cytomics FC 500 flow cytometer (Beckman Coulter, Fullerton, CA). All experiments were performed at least three times.

### Statistical Analysis

All data are expressed as mean ± standard deviation. Differences between two groups were determined using Student’s t tests. All statistical analyses were conducted with SPSS 19.0 software (SPSS, Inc., Chicago, IL). A difference was considered statistically significant at *p* < 0.05.

## Additional Information

**How to cite this article**: Rong, X. *et al.* Effects of histone acetylation on superoxide dismutase 1 gene expression in the pathogenesis of senile cataract. *Sci. Rep.* **6**, 34704; doi: 10.1038/srep34704 (2016).

## Supplementary Material

Supplementary Information

## Figures and Tables

**Table 1:** Baseline characteristics of the patient samples.

Category	Control	NC	CC	SC
Eyes (n)	12	40	40	40
Male	7	18	19	20
Female	5	22	21	20
Age (years)	35.3 ± 2.8	74.9 ± 7.6	70.2 ± 9.9	71.4 ± 9.1
Axial length (mm)	22.89 ± 0.64	22.96 ± 0.76	23.07 ± 0.77	23.08 ± 0.60
