# Supplementary material for: Effects of histone acetylation on superoxide dismutase 1 gene expression in the pathogenesis of senile cataract
Source: Sci Rep. 2016 Oct 5;6:34704. doi: 10.1038/srep34704 (PMC5050424; doi:10.1038/srep34704)
Supplement: Supplementary Information [file srep34704-s1.doc]

# Effects of histone acetylation on superoxide dismutase 1 gene expression in the pathogenesis of senile cataract

Xianfang Rong#, Xiaodi Qiu#, Yongxiang Jiang, Dan Li, Jie Xu, Yinglei Zhang, Yi Lu*

Department of Ophthalmology, Eye and ENT Hospital of Fudan University, Shanghai, China; Key Laboratory of Myopia of State Health Ministry, and Key Laboratory of Visual Impairment and Restoration of Shanghai, Shanghai, China.

Xianfang Rong and Xiaodi Qiu contributed equally to this work.

*Corresponding author: Yi Lu, Department of Ophthalmology, Eye and ENT Hospital of Fudan University, No.83 Fenyang Road, Shanghai, China, 200031. Email: [luyieent@126.com](mailto:luyieent@126.com)

Supplementary Table S1. Sequences of the primers designed for quantitative real-time PCR.

| Primers | Human sequences | Rabbit sequences |
| --- | --- | --- |
| SOD1 | 5’-GGGCAATGTGACTGCTGAC-3’ | 5’-AAGGGACGCATAACAGGACT-3’ |
|  | 5’-ACAAGCCAAACGACTTCCAG-3’ | 5’-AACACATCAGCGACACCATT-3’ |
| β-actin | 5’-CCCTGGACTTCGAGCAAGAG-3’ | 5’-CGGGACATCAAGGAGAAGC-3’ |
|  | 5’-TCACACTTCATGATGGAGTTG-3’ | 5’-AGGAAGGAGGGCTGGAAGA-3’ |

Supplementary Table S2. Sequences of oligonucleotide primers used in CHIP-PCR assay

| Promoter region of SOD1 | Sequences in cataract patients | Sequences in rabbits |
| --- | --- | --- |
| -3000 | 5’-AAGTGCAAAGGTCCCTCTCA-3’ | 5’-GACTTGGTAATGTATGGAGA-3’ |
|  | 5’-TAAGACAGGATGATAGAGCC-3’ | 5’-CAGCATCTGCCCCATGAAGC-3’ |
| -2700 | 5’-GGCTCTATCATCCTGTCTTA-3’ | 5’-GCTTCATGGGGCAGATGCTG-3’ |
|  | 5’-AACCACTGTTTAGAACACCA-3’ | 5’-ACAAATCCCAGCCAAAGGGC-3’ |
| -2400 | 5’-TGGTGTTCTAAACAGTGGTT-3’ | 5’-GCTTATGTAATTCTTCCGTA-3’ |
|  | 5’-TTTATGTATTTATTTTGATA-3’ | 5’-TTACAACTCGCTAGGCAGTG-3’ |
| -2100 | 5’-TATCAAAATAAATACATAAA-3’ | 5’-GAGTGACTTCTACTCCCAGG-3’ |
|  | 5’-TCCCCCCGCAAGAATATCAT-3’ | 5’-TCAGTGTTCTTAACCGCTGA-3’ |
| -1800 | 5’-ATGATATTCTTGCGGGGGGA-3’ | 5’-CTGGTCTTCCAGAGGTCCTG-3’ |
|  | 5’-ACTGGGACTTTCTCGGAGCG-3’ | 5’-GCATAAAACTGCATGTTCTG-3’ |
| -1500 | 5’-CGCTCCGAGAAAGTCCCAGT-3’ | 5’-ATGAGCTCTTGGGAGACCAC-3’ |
|  | 5’-CAAAGGGCCTTTGCCCAAGG-3’ | 5’-TAAGACGGTATGGAATGAGC-3’ |
| -1200 | 5’-CCTTGGGCAAAGGCCCTTTG-3’ | 5’-GAAAATATCTTCCTTGAAGC-3’ |
|  | 5’-AGTACTTAATATTGAGTATG-3’ | 5’-TCTCCCTGCCTCTGCCTCTG-3’ |
| -900 | 5’-CATACTCAATATTAAGTACT-3’ | 5’-GCCCTGTGAGTTTGAGGTCA-3’ |
|  | 5’-GTATAGAGTTTCTTATTTGG-3’ | 5’-GCTGGGGAATCGAACCCAGG-3’ |
| -600 | 5’-CCAAATAAGAAACTCTATAC-3’ | 5’-TCCGAAAAAAAGAACCCCCC-3’ |
|  | 5’-AAACAACCTTCTTTTCACGG-3’ | 5’-AAGTCGCTATTGTCTTCTA-3’ |
| -300 | 5’-CCGTGAAAAGAAGGTTGTTT-3’ | 5’-TCCCAGCTCTGTCTCGATTC-3’ |
|  | 5’-AACTCGCTAGGCCACGCCGA-3’ | 5’-CGCCATGCTTCCCCGGGAG-3’ |

Supplementary Figure S1


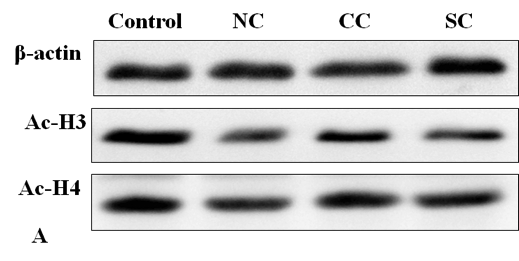

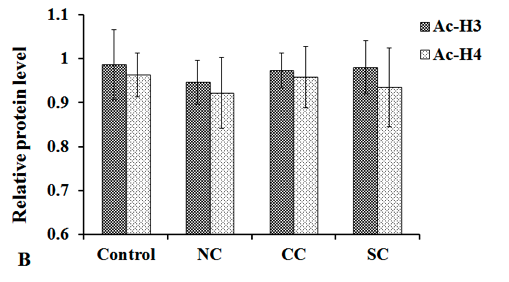


Western blot analysis of genome-wide histone acetylation in senile cataracts. (A) Acetylation levels of H3 and H4 were measured. β-Actin was used as a protein loading control. (B) Acetylation levels of H3 and H4 were quantitatively analyzed relatively to β-actin. As shown, although a slight decrease in acetylation was detected in H3 and H4 of the cataract lenses, no significant differences were found between the NC, CC, and SC groups compared with the control group.

Supplementary Figure S2


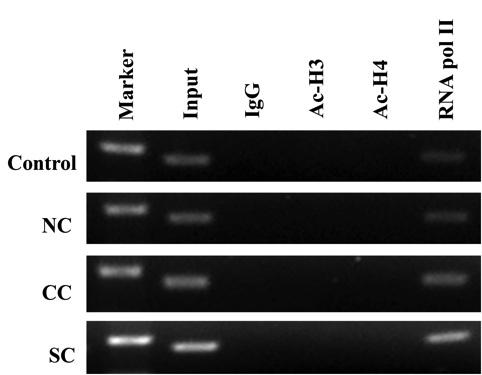


PCR analysis of DNA in Input and DNA precipitated by IgG, ac-H3 and ac-H4 antibodies as well as RNA pol II antibody in human LE. In order to prove the effectiveness and reliability of the CHIP-PCR anaysis reults of human LE sapmles in the control, NC CC and SC, the DNA precipitated by IgG, ac-H3 and ac-H4 antibodies as well as RNA pol II antibody was underwent PCR assay by human GAPDH promoter primes. As shown in the figure above, positive bands were presented in Input and DNA precipitated by RNA pol II antibody. While the results of the DNA precipitated by IgG, ac-H3 and ac-H4 antibodies showed negative. (Sequences of human oligonucleotide primers used in PCR assay: 5’-GGCTGGGACTGGCTGAGCCT-3’, 5’-TCGAACAGGAGGAGCAGAGA-3’)

Supplementary Figure S3


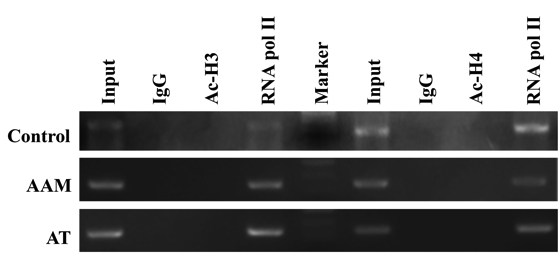


PCR analysis of DNA in Input and DNA precipitated by IgG, ac-H3 and ac-H4 antibodies as well as RNA pol II antibody in rabbit LE. In order to prove the effectiveness and reliability of the CHIP-PCR anaysis reults of rabbit LE sapmles in the control, AAM and AT groups, the DNA precipitated by IgG, ac-H3 and ac-H4 antibodies as well as RNA pol II antibody was underwent PCR assay by human GAPDH promoter primes. As shown in the figure above, positive bands were presented in Input and DNA precipitated by RNA pol II antibody. While the results of the DNA precipitated by IgG, ac-H3 and ac-H4 antibodies showed negative. (Sequences of rabbit oligonucleotide primers used in PCR assay: 5’-CCCGTTCGACAGGCAGCC-3’, 5’- CGTGTCTCGGGGACGCGC-3’)

Supplementary Figure S4


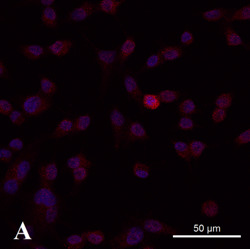

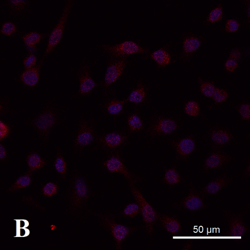

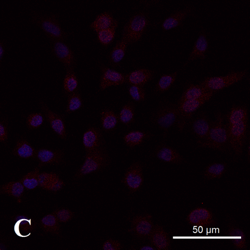


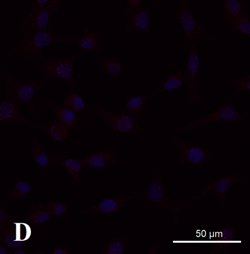

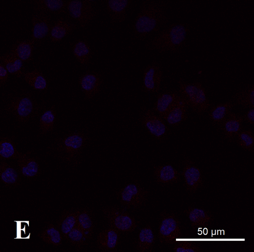

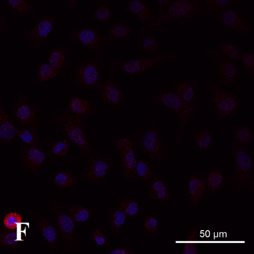


Immunofluorescence staining of SOD1 expressed in HLECs. The immunofluorescence intensity of SOD1 is remarkably weaker in Figures D and E. In Figure F, the stained SOD1 is almost the same intensity as in Figure D. A) control group; B) DMSO group; C) AAL group; D) AAM group; E) AAH group; F) AT group.

Supplementary Figure S5


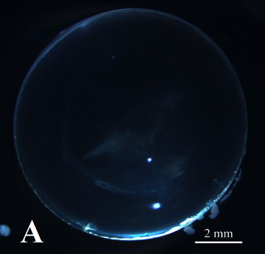

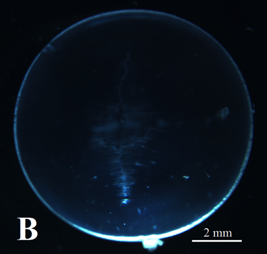

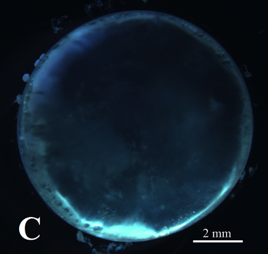


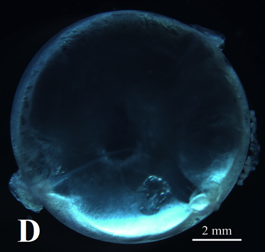

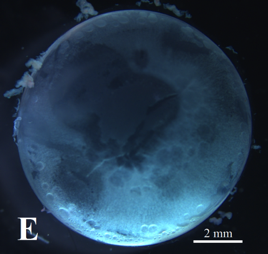

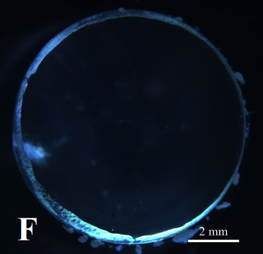


Rabbit lens culture supplemented with different concentrations of AA and AA+TSA for three weeks. The lenses developed opacification in a concentration-dependent fashion. TSA maintained the transparency of the lenses in the AT group, except for the opacification that formed in the equatorial area. A) control group; B) DMSO group; C) AAL group; D) AAM group; E) AAH group; F) AT group.
